# GluN2D NMDA Receptors Gate Fear Extinction Learning and Interneuron Plasticity

**DOI:** 10.3389/fnsyn.2021.681068

**Published:** 2021-05-24

**Authors:** Christophe J. Dubois, Siqiong June Liu

**Affiliations:** ^1^Department of Cell Biology and Anatomy, LSU Health Sciences Center New Orleans, New Orleans, LA, United States; ^2^Southeast Louisiana VA Healthcare System, New Orleans, LA, United States

**Keywords:** GluN2D NMDA receptor, fear conditioning, extinction learning, metaplasticity, long-term depression, inhibitory synapses, D-cycloserine, cerebellar molecular layer interneurons

## Abstract

The cerebellum is critically involved in the formation of associative fear memory and in subsequent extinction learning. Fear conditioning is associated with a long-term potentiation at both excitatory and inhibitory synapses onto Purkinje cells. We therefore tested whether fear conditioning unmasks novel forms of synaptic plasticity, which enable subsequent extinction learning to reset cerebellar circuitry. We found that fear learning enhanced GABA release from molecular layer interneurons and this was reversed after fear extinction learning. Importantly an extinction-like stimulation of parallel fibers after fear learning is sufficient to induce a lasting decrease in inhibitory transmission (I-LTD_stim_) in the cerebellar cortex, a form of plasticity that is absent in naïve animals. While NMDA (N-methyl-D-aspartate) receptors are required for the formation and extinction of associative memory, the role of GluN2D, one of the four major NMDA receptor subunits, in learning and memory has not been determined. We found that fear conditioning elevates spontaneous GABA release in GluN2D KO as shown in WT mice. Deletion of GluN2D, however, abolished the I-LTD_stim_ induced by parallel fiber stimulation after learning. At the behavioral level, genetic deletion of GluN2D subunits did not affect associative learning and memory retention, but impaired subsequent fear extinction learning. D-cycloserine, a partial NMDA receptor (NMDAR) agonist, failed to rescue extinction learning in mutant mice. Our results identify GluN2D as a critical NMDAR subunit for extinction learning and reveal a form of GluN2D-dependent metaplasticity that is associated with extinction in the cerebellum.

## Introduction

Pavlovian fear learning is one of the best-characterized model systems of emotional memory in which an individual learns to associate a neutral stimulus with an aversive event. This form of associative memory can be attenuated following repetitive exposures to the neutral stimulus, producing extinction learning (Dunsmoor et al., 2015), a strategy that has been used for the treatment of anxiety and post-traumatic stress disorders (Bowers and Ressler, 2015). Our understanding of the molecular mechanism and the neuronal basis of extinction of fear memory is therefore of prime importance, but remains incomplete at present.

Fear conditioning alters synaptic transmission in a number of brain regions that are important for memory consolidation (Izquierdo et al., 2016; Bocchio et al., 2017), including the prefrontal cortex, hippocampus, and the amygdala (Apps and Strata, 2015; Tovote et al., 2015). In addition to these extensively studied neuronal circuits, clinical studies implicate the cerebellum in emotional regulation and fear memory extinction (Timmann et al., 2010; Linnman et al., 2011; Lange et al., 2015; Utz et al., 2015; Ernst et al., 2019). The cerebellum is required for associative fear memory formation, as reversible inhibition of neuronal activity or release endocannabinoids from Purkinje cells after learning disrupts memory consolidation (Sacchetti et al., 2002; Dubois et al., 2020). Associative fear learning enhances both excitatory (Sacchetti et al., 2004) and inhibitory (Scelfo et al., 2008) transmission to Purkinje cells, and reduces endocannabinoid signaling, increasing GABA release (Dubois et al., 2020). Molecular layer interneurons (MLIs) in the cerebellar cortex control the activity of Purkinje cells and thus a learning-induced enhancement of GABA release from MLIs can alter the activity and output of cerebellar circuitry (Scelfo et al., 2008). A recent study shows that the cerebellum to the ventrolateral periaqueductal gray projection regulates extinction learning (Frontera et al., 2020), indicating that the cerebellum is involved in both fear memory formation and subsequent extinction learning. We hypothesize that learning experience allows the cerebellar circuit to undergo novel form of synaptic plasticity that is absent in the naive animals, and thereby to engage in new extinction learning.

Learning-induced changes in synaptic transmission and plasticity are cellular mechanisms that underlie the formation and subsequent extinction of fear memory. Considerable evidence suggest that NMDA-dependent plasticity is a key component of the extinction learning processes (Sotres-Bayon et al., 2007; Davis, 2011; Ogden et al., 2014). NMDA receptors are tetrameric glutamate receptors composed of two GluN1 subunits associated with GluN2/3 (GluN2A–2D; GluN3A–3B) subunits. Of the four GluN2 subunits, GluN2D subunits are expressed at high levels in inhibitory interneurons and exhibit two distinct properties, very high affinity for glutamate and exceedingly slow deactivation time course (Cull-Candy and Leszkiewicz, 2004). Thus these receptors are capable of detecting low levels of spillover glutamate and play an important role in the plasticity of interneurons (Hunt and Castillo, 2012; Paoletti et al., 2013). D-cycloserine, a partial NMDAR agonist has been shown to promote extinction in rodents (Walker et al., 2002; Ledgerwood et al., 2005; Mao et al., 2006) and has a higher binding affinity for receptors that contain GluN2C and GluN2D subunits than other subunits (Sheinin et al., 2001; Dravid et al., 2010). This is important because potentiation of GluN2C/2D-containing NMDARs with CIQ ((3-Chlorophenyl) [3,4-dihydro-6,7-dimethoxy-1-[(4-methoxyphenoxy)methyl]-2(1H)-isoquinolinyl]methanone) is sufficient to promote extinction learning (Ogden et al., 2014). While deletion of GluN2C leads to a deficit in associative fear learning (Hillman et al., 2011), inhibition of GluN2B-containing receptors impairs extinction of fear memory (Sotres-Bayon et al., 2007). Surprisingly, GluN2D function in behavior remains unknown, largely due to the lack of selective inhibitors. Given the unusual high sensitivity of GluN2D-containing NMDA receptors for glutamate and D-cycloserine and their unique involvement in synaptic plasticity, we tested the hypothesis that GluN2D-containing NMDARs are required for the extinction of fear memory and extinction learning-induced synaptic plasticity in cerebellar interneurons.

Here we show that fear conditioning induced a lasting increase in GABA release from cerebellar MLIs and extinction learning reduced inhibitory transmission. After fear conditioning, stimulation of cerebellar parallel fibers (the axons of granule cells, PFs) using a protocol that mimics extinction learning, induced a lasting suppression of GABA release (I-LTD_stim_), and genetic deletion of GluN2D abolished I-LTD_stim_. This form of plasticity occurs only in conditioned mice and requires GluN2D-containing NMDARs. At behavioral level GluN2D knockout mice exhibited impaired extinction learning and memory. Furthermore, D-cycloserine and retrieval session, which accelerated extinction learning in wildtype mice, no longer enhanced extinction in GluN2D knockout mice. Therefore GluN2D is crucial for extinction learning and associated synaptic plasticity.

## Materials and Methods

### Animals

Male mice on a C57Bl/6J background were used for this study. These animals were either wildtype (Jackson laboratory Bar Harbor, ME, United States) or GluN2D KO mice (Ikeda et al., 1995; Dubois et al., 2016). Breeding colonies were maintained in our animal facility on a 12h light/dark cycle, with *ad libitum* food and water. Genotyping was performed by Mouse Genotype^1^, using a common forward sequence GCAGGCCCCTGCCTCCTCGCTC, a reverse GluN2D KO primer sequence TGGATTGCACGCAGGTTCTC, and a reverse wild-type primer sequence CTGACCTCATCCTCAGATGAG generating a PCR product of 982 bp for GluN2D KO, and 281 bp for GluN2D wildtype. Experimental procedures were in accordance with the US National Research Council’s Guide for the Care and Use of Laboratory Animals and approved by the Louisiana State University Health Sciences Center guidelines for care and use of laboratory animals (IACUC).

### Fear Conditioning Apparatus

*Context A* Fear conditioning experiments were conducted in a non-reflective black box with 28 × 28 × 30 cm dimensions. Stainless steel rods (spaced at 0.5 cm) delivered a 0.75 mA foot-shock (unconditioned stimulus, United States) via a shock delivery apparatus (Model H13–15, Coulbourn Instruments, Holliston, MA, United States). The conditioned stimulus (CS) was a 3.5 kHz sound at 75 dB delivered through a 75 mm speaker. The conditioning apparatus was placed in a sound–reducing chamber (typical background noise was 65 dB). The timing and length of both the CS and US were set using custom software written by Dr. Iaroslav Savtchouk (Marquette University).

*Context B* The memory retention test and extinction training were conducted in a plastic chamber (20 × 35 × 40 cm) with off-white color walls and white paper bedding covering the floor. The speaker was positioned at a different location relative to context A to deliver the CS. The apparatus was placed in the sound–reducing chamber.

### Fear Conditioning Procedure

Experiments took place at the beginning of the dark phase. The experimenter was blind to the genotype of the animals at the time of the test. All animals were identified by marks on the tail and weighed 1 h before the conditioning session on both days. All experiments were video-recorded (Windows Media Encoder, Microsoft) and stored on a computer for off-line analysis.

#### Fear Conditioning (Context A)

On day one, 2–3 months-old males were positioned in the center of the arena and allowed to explore for 2 min. Conditioning consisted of two or eight pairings of a 10 s sound (CS) co-terminated with a 1 s foot-shock (US). Each pairing started 30 s apart. After a 2 min recovery period, animals were returned to their home cage until the next day.

#### Drug Injections

In some experiments, mice were injected i.p 30 min prior to cued memory retention and extinction learning testing. After saline injections all tested parameters were identical to non-injected animals. Therefore, results from saline-injected and non-injected animals were pooled for presentation of D-cycloserine experiments.

#### Cued Memory Retention and Extinction Learning (Context B)

These procedures were conducted on day 2 in Context B and mice were positioned in the center of the arena. When conditioned with two pairings of US/CS, after a 2 min habituation period, 8 CS of 10 s were presented every 30 s. When conditioned with eight pairings, mice were first exposed to a retrieval CS in context B, following a 2 min acclimation period, and then returned to their home cage for 30 min. Mice were then exposed to a series of twenty 10 s CS every 30 s and a second extinction session of 20 CS, 30 min later.

#### Extinction Retention (Context B)

Following two extinction sessions, mice were tested for extinction retention with 4 CS exposure every 30 s on day 3.

#### Behavioral Quantification

A freezing (immobility) episode was defined as a complete absence of movement apart from respiratory activity for at least 1 s. This was characterized by the amount of motion that occurred between two successive video frames, using a custom-written program previously described (Liu et al., 2010). The duration of freezing responses was determined during the 2 min of habituation and during the first 9 s of each tone.

### Behavioral Procedures for Electrophysiology

All conditioning procedures were conducted during the dark phase of the light/dark cycle, about 15 h before slice preparation. Male mice were submitted to a conditioning procedure that was identical to the one described for behavioral testing, with eight pairings of CS/US. To ensure a slice quality that would allow us to conduct stable patch-clamp recordings for over an hour, 3–5 weeks old mice were used for electrophysiology experiments. Although at this age mice were less spontaneously active, they exhibited fear learning (41% increase from basal freezing; *n* = 45, data not shown) and extinction (36% decrease, from tone 1 to tone 8, *n* = 17, data not shown). Furthermore, the frequency of IPSCs and synaptic plasticity in stellate cells was not different from 2 to 3 months old mice (18–33 days-old mice IPSC frequency 5.3 ± 0.6 Hz, *n* = 17; 46–90 days-old mice IPSC frequency 5.3 ± 1.7 Hz, *n* = 5; two-sided *P* value of the Mann–Whitney test is 0.959, data not shown).

### Cerebellar Slice Preparation and Electrophysiology

Cerebellar slices were prepared as previously described (Liu and Cull-Candy, 2000; Dubois et al., 2016). Briefly, the cerebellum was isolated and horizontal slices (400 μm) were cut using a vibratome (Leica VT1200) in ice cold artificial CSF (containing in mM: 81.2 NaCl, 2.4 KCl, 23.4 NaHCO_3_, 1.4 NaH_2_PO_4_, 6.7 MgCl_2_, 0.5 CaCl_2_, 23.3 glucose, 69.9 sucrose, and pH 7.4). Slices were then maintained in aCSF (in mM: 125 NaCl, 2.5 KCl, 26 NaHCO_3_, 1.25 NaH_2_PO_4_, 1 MgCl_2_, 2 CaCl_2_, 25 glucose, and pH 7.4) saturated with 95% O_2_, 5% CO_2_ at room temperature for at least 30 min before recording.

Whole cell patch clamp recordings were obtained at near physiological temperature (35–37°C) from cerebellar stellate cells in an O_2_/CO_2_-saturated aCSF. Stellate cells were identified by their location in the outer two thirds of the molecular layer and by the presence of spontaneous action potentials in the cell-attached mode. Analog signals were filtered at 6 kHz and digitized at 20 kHz (Multiclamp 700A, Axon Instruments). Series resistance was monitored throughout the recordings. Recordings were terminated if series resistance changed by more than 20%.

### Long-Term Depression of Inhibitory Transmission

**FIGURE 1:**
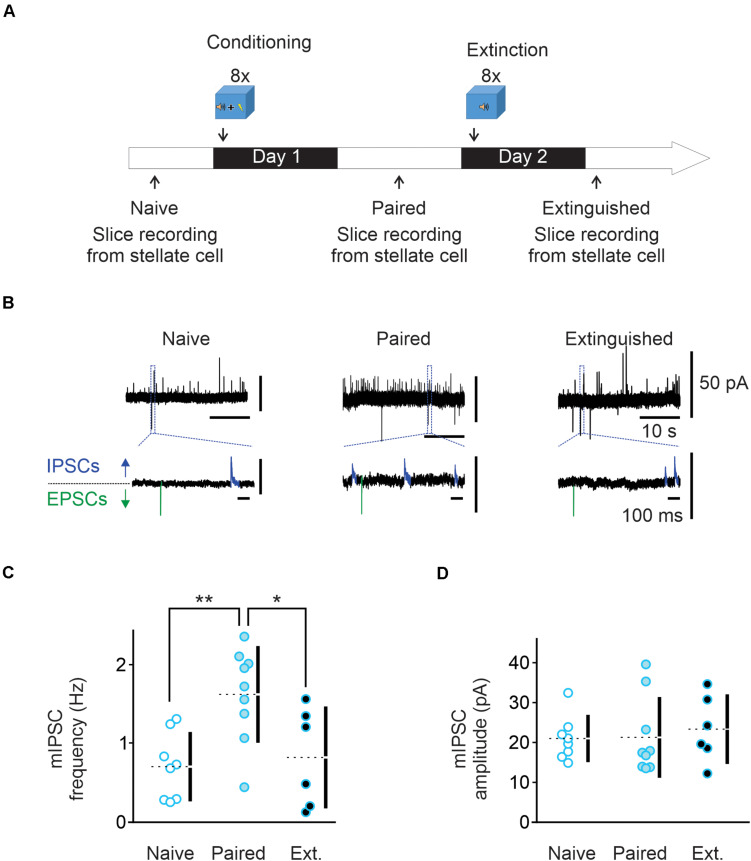
Fear conditioning enhances and extinction learning reduces spontaneous GABA release from cerebellar molecular layer interneurons. **(A)** Conditioning protocol. Male mice (*n* = 9) were habituated for 2 min in the conditioning chamber (context A) and exposed to eight pairings of a 10 s tone that co-terminated with a 1 s foot-shock (paired animals). Mice were left in the conditioning chamber for another 2 min before being returned to their home cage. Cerebellar slices were prepared and electrophysiology experiments were performed 15 h later. A subset of Extinguished mice (*n* = 6) were exposed to eight tones alone 24 h after fear conditioning protocol. Cerebellar slices were prepared and electrophysiology experiments were performed 15 h later. **(B)** Representative traces recorded in putative stellate cells from the three behavioral groups at -30 mV. The *bottom* traces are enlargements of regions designated by the dashed lines. In these traces, outward IPSCs are highlighted in blue while inward EPSCs are shown in green. **(C,D)** Individual frequency and amplitude of the recorded mIPSCs from the three behavioral groups. Mean values are represented as doted lines. *P < 0.05; **P < 0.01. Statistical analysis values can be found in the Supplementary Table 1.

Miniature inhibitory synaptic currents (mIPSCs) were recorded in stellate cells in the presence of 0.5 μM TTX (Tetrodotoxin) in aCSF, using borosilicate electrodes (6–8 MΩ) filled with a low chloride pipette solution (in mM: 120 Cs acetate, 0.4 MgCl_2_, 0.1 CaCl_2_, 2.5 MgATP, 0.4 Na_2_GTP, 1.5 Na_2_ATP, 10 Cs-EGTA, 5 QX-314 and 10 HEPES, and pH 7.3). Using this internal solution, when putative stellate cells were voltage-clamped at –30 mV, the chloride-mediated mIPSCs were recorded as outward currents (blue events on Figure 1B) whereas cation-mediated mEPSCs appeared as inward currents (green events in Figure 1B), allowing for separation of IPSCs from EPSCs without the use of pharmacological agents. Indeed, pharmacologically blocking glutamatergic neurotransmission to isolate mIPSC would interfere with activation of NMDA receptors during the parallel fiber stimulation. After at least 15 min of stable recording (control period), TTX was washed out for 20 min. Parallel fibers were then stimulated using a parallel bipolar electrode (150 μm branch spacing, 200 μm from the recording electrode). The stimulation strength was adjusted to evoke NMDA receptor currents at +40 mV in response to a single burst stimulation (four stimuli at 100 Hz) and ranged from 5 to 45 V (200 μs duration). I-LTD_stim_ was then induced using 15 trains of burst stimulation (four stimuli at 100 Hz repeated every second for 15 s). The postsynaptic cell was voltage-clamped at –60 mV during the parallel fiber stimulation. TTX was re-introduced into the aCSF and recordings of mIPSCs were resumed within 2 min and lasted for 30–50 min.

### Data Analysis

Clampfit 9.0 (Axon Instruments) was used for mIPSCs analysis using an event detection template. The average frequency and amplitude were calculated over periods of 5 min. Miniature EPSC frequency was very low as granule cells were not spontaneously active, and therefore was not quantified.

No statistical method was used to predetermine sample sizes, but they are similar to previous studies (Liu and Cull-Candy, 2000; Lachamp et al., 2009; Dubois et al., 2016, 2020). Each data set was obtained from mice from at least three different litters. Statistical analyses were performed using the estimation statistics webpage^2^. Mann–Whitney test was used to test for significance between the means of two independent groups. Comparison between the means of three or more independent groups of normally distributed data was conducted using a one-way analysis of variance (ANOVA). A two-way ANOVA was used to test for an interaction between two independent variables and the dependent variable. The paired-sample Wilcoxon test was used to test for significance between the amplitude and frequency of miniature inhibitory currents recorded before and after synaptic stimulation. All values are represented as mean ± SEM and a *P* value of 0.05 was considered as significant. All tests were performed on primary data (not normalized). For detailed statistical analysis, see the Supplementary Table 1. Data will be available upon request from the corresponding author.

## Results

### Fear Conditioning Increases Spontaneous GABA Release, and This Is Reversed by Extinction Training

Fear conditioning has been shown to induce a lasting increase in spontaneous GABA release onto Purkinje and MLIs (Scelfo et al., 2008; Dubois et al., 2020). We determined the effects of learning and extinction paradigms on GABA release from MLIs to synaptic connected MLIs. Animals were subject to eight pairs of a tone co-terminated with a mild electric footshock (paired group). Cerebellar slices were prepared from naïve and conditioned mice next day (Figure 1A). Miniature IPCSs were recorded at –30 mV in the presence of 0.5 μM TTX in putative stellate cells in the vermal lobules V/VI, where nociceptive and acoustic stimuli converge in the cerebellum. Using a low chloride pipette solution, mIPSCs were recorded as outward currents (blue in Figure 1B) and mEPSCs as inward currents (green in Figure 1B). In MLIs from naïve mice, the average mIPSC frequency was 0.69 ± 0.15 Hz and amplitude was 20 ± 2 pA (*n* = 8, Figures 1B–D). After fear conditioning, the average mIPSC frequency was increased to 1.62 ± 0.2 Hz (*n* = 9, Figures 1B,C) while the amplitude remained unchanged at 21 ± 3 pA (Figures 1B,D). These results suggest that fear conditioning increases spontaneous GABA release, but not the postsynaptic response, consistent with previous observation in Purkinje cells (Scelfo et al., 2008).

Next, a group of animals were exposed to a series of eight tones without footshock in a novel environment one day after fear conditioning paradigm (Extinction group) as depicted in Figure 1A. Cerebellar slices were prepared 15 h later. We found that mIPSC frequency was markedly reduced compared to the paired group (I-LTD_*ext*_, 0.81 ± 0.25 Hz, *n* = 6; Figures 1B,C), and was comparable to that obtained in naïve animals. The amplitude of mIPSCs was unchanged compared to naïve and paired groups, with an average value of 23 ± 3 pA (Figure 1D). Therefore, extinction learning reverts the learning-induced increase in spontaneous GABA release to a level indistinguishable from the naïve state.

### An Extinction-Like Stimulus After Fear Conditioning Induces a Lasting Decrease in GABA Release, I-LTD_stim_

In the cerebellar cortex, acoustic stimulation activates parallel fibers (Aitkin and Boyd, 1978), and therefore the CS (*i.e*., tones) during the extinction protocol, is expected to stimulate these excitatory inputs. We tested whether stimulation of parallel fibers in slices prepared from conditioned mice can induce a lasting decrease in GABA release, to account for the reduction observed after *in vivo* extinction learning.

**FIGURE 2:**
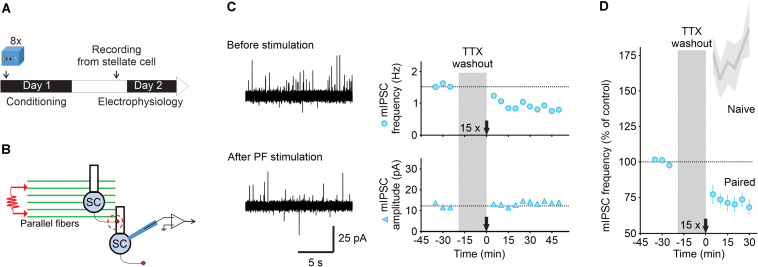
Parallel fiber stimulation triggers a long-lasting decrease in GABA release after fear learning. **(A)** Fear conditioning protocol for electrophysiology experiments. **(B)** Schematic of the experimental procedure. **(C)** Left, representative traces of mIPSCs recorded in a stellate cell at -30 mV (outward currents) before (top) and after (bottom) PF stimulation (15 trains of four pulses at 100 Hz). Right, corresponding time course of mIPSC frequency (top) and amplitude (bottom). **(D)** While this protocol triggered I-LTP in naïve wildtype animals [Shaded gray represent data from Dubois et al. (2016)], after fear conditioning burst activation of parallel fibers induced I-LTD_stim_ (*n* = 5). Values are mean ± SEM. Statistical analysis values can be found in the Supplementary Table 1.

Slices were obtained 15 h after fear conditioning (Figure 2A). We recorded mIPSCs in stellate cells in the presence of 0.5 μM TTX to assess spontaneous GABA release in lobules V/VI. After obtaining a stable baseline, TTX was washed out for 20 min and we stimulated parallel fibers with 15 trains of four stimulations at 100 Hz repeated at 1 Hz (Figure 2B). TTX was then re-introduced and recording of mIPSCs was resumed for 30 min or longer. We found that parallel fiber stimulation induced a rapid decrease in the mIPSC frequency from 1.5 ± 0.15 to 1.04 ± 0.14 Hz (*n* = 5, Figures 2C,D), a level that is comparable with the one observed after *in vivo* extinction learning. This reduction in mIPSC frequency was observed in all cells recorded and lasted for at least 30–50 min after stimulation without changing the mIPSC amplitude. These results suggest that stimulation of parallel fibers in slices from conditioned animals triggers a lasting decrease in GABA release from MLIs (I-LTD_stim_, parallel fiber stimulation-induced long-term depression at inhibitory synapses) that mimics the I-LTD_*ext*_ observed after extinction of associative fear learning.

Learning can unmask novel forms of synaptic plasticity that are absent in naïve animals, inducing a form of metaplasticity, that enables subsequent experience such as extinction to reset synaptic transmission (Hulme et al., 2013). Our previous work has shown that stimulation of parallel fibers induces a lasting increase in GABA release from stellate cells in naïve mice (I-LTP, Dubois et al., 2016). However, after fear conditioning it induced a sustained decrease in GABA release from cerebellar interneurons (I-LTD_stim_). Therefore, fear conditioning enables the parallel fiber stimulation to induce I-LTD_stim_, a stimulus that produces I-LTP in naïve mice, giving rise to a form of metaplasticity.

### I-LTD_stim_ Requires GluN2D-Containing NMDA Receptors

We have previously shown that MLIs, like many other inhibitory interneurons, express GluN2D subunits. In naïve mice GluN2D and GluN2B subunits form tri-heteromeric receptors (GluN1/2B/2D) and activation of these receptors induces I-LTP (Dubois et al., 2016). Extinction learning is known to require activation of NMDA receptors, but the role of GluN2D in extinction is not known. We therefore tested the role of GluN2D in I-LTD_stim_ induced by the extinction-like stimulus after fear conditioning.

**FIGURE 3:**
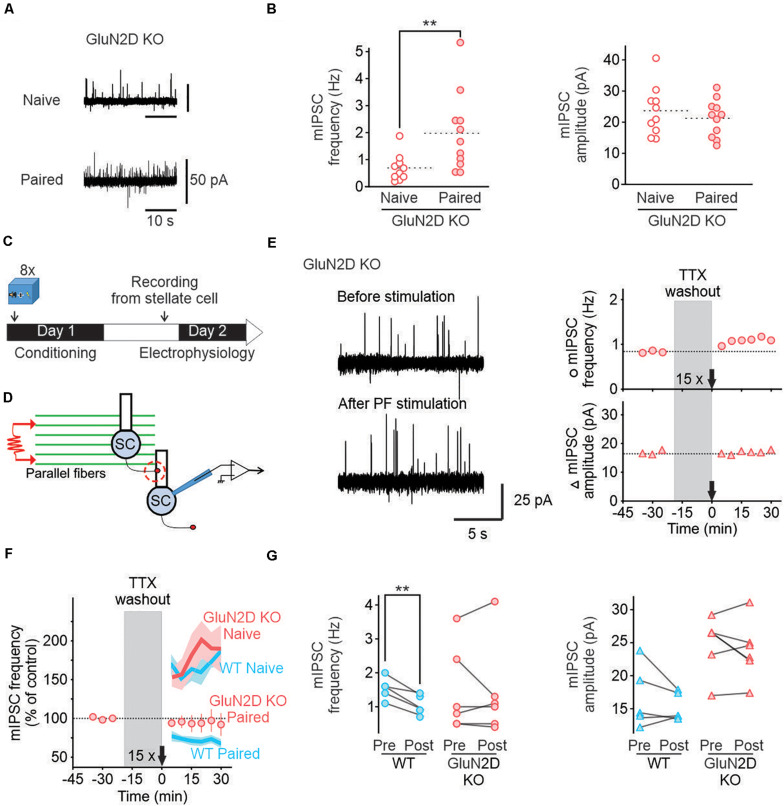
Deletion of GluN2D subunits abolishes I-LTD_stim_ in conditioned mice. **(A)** Representative traces of mIPSCs in stellate cells from naïve (*top*) and paired (*bottom*) GluN2D KO mice. **(B)** Individual frequencies and amplitudes of mIPSCs in naïve (open red circle, *n* = 10) and paired mutant animals (filled red circles, *n* = 11). **(C)** Fear conditioning protocol for electrophysiology experiments. **(D)** Schematic of the experimental procedure. **(E)** *Left*, Example traces of mIPSCs recorded in MLIs before (*top*) and after (*bottom*) parallel fiber stimulation (15 trains of four pulses at 100 Hz) in GluN2D KO mice after fear conditioning. *Right*, corresponding time course of mIPSC frequency (top) and amplitude (bottom). **(F)** Average time course of mIPSC frequency normalized to before parallel fiber stimulation in GluN2D KO mice (red circles, values are mean ± SEM) after fear learning. Wildtype naïve and paired group average values (blue lines) and SEM (light blue area) are data from Figure 3 represented for reference. **(G)** Summary of the individual frequencies (*left*) and amplitudes (*right*) before (*Pre*) and 15–30 min after parallel fiber stimulation (*Post*). **P < 0.01. Statistical analysis values can be found in the Supplementary Table 1.

We used homozygous GluN2D knockout mice in which no GluN2D protein was detected (Ikeda et al., 1995). Deletion of GluN2D abolished characteristic low conductance currents in single channel recording and reduced NMDAR-current decay time, indicating a loss of functional GluN2D subunits in these neurons (Dubois et al., 2016). We have previously shown that deletion of GluN2D did not alter glutamate release from parallel fibers, nor spontaneous GABA release from MLIs in naïve mice (Dubois et al., 2016). First, we tested whether learning still can increase GABA release in GluN2D KO mice. The average mIPSC frequency in naïve GluN2D KO mice was 0.68 ± 0.16 Hz with an amplitude of 24 ± 2 pA (*n* = 10, Figures 3A,B), comparable with that in naïve wildtype mice. One day after fear conditioning acquisition mIPSCs frequency in MILs rose to 1.96 ± 0.44 Hz (*n* = 11, Figures 3A,B) while the amplitude remained unaltered (22 ± 2 pA). This increase in spontaneous mIPSC frequency was similar to that observed in wildtype mice after conditioning. Thus deletion of GluN2D did not affect basal and learning-induced change inhibitory transmission.

We next investigated the role of GluN2D-containing receptors in I-LTD_stim_ after fear conditioning using GluN2D KO mice (Figures 3C,D). In contrast to wildtype mice, activating parallel fibers with 15 trains of stimuli after fear conditioning failed to reduce the frequency (1.42 ± 0.56 Hz, *n* = 6, Figures 3E–G) or amplitude of mIPSCs recorded in MLIs (24 ± 2 pA, Figures 3E–G). The failure to induce I-LTD_stim_ in GluN2D KO mice is unlikely to be due to a change in GABA release since deletion of GluN2D did not alter GABA release in naïve and conditioned mice (Dubois et al., 2016 and Figures 1C, 2B). Therefore, GluN2D-containing NMDA receptors are critical for the induction of I-LTD_stim_. Because the 15 train-parallel fiber stimulation induced I-LTP in naïve mutant animals (Dubois et al., 2016), learning unmasks a novel role of GluN2D in regulating GABA release from MLIs.

### Genetic Deletion of GluN2D-Containing NMDA Receptors Abolishes Fear Extinction Learning

NMDA receptor-dependent neuronal plasticity is a key component of the extinction learning process (Sotres-Bayon et al., 2007; Davis, 2011; Ogden et al., 2014). We confirmed NMDA receptors mediates extinction learning as administration of memantine (5 mg/kg, i.p.), an NMDAR inhibitor (Bresink et al., 1996), 30 min before extinction abolished extinction learning in wildtype mice (Supplementary Figures 3A–C) without a change in learning acquisition (Supplementary Figures 1A,C). Thus activation of NMDA receptors is required for fear extinction learning in our paradigm. While GluN2B-containing receptors contribute to extinction of fear memory (Sotres-Bayon et al., 2007), the role of GluN2D in learning and memory remains unknown. Our results so far show that fear conditioning increases GABA release from MLIs. When exposed *in vivo* to an extinction protocol, GABA release returns to pre-conditioning levels (I-LTD_*ext*_). This LTD of inhibitory synapses can be mimicked *in vitro* with an extinction-like stimulation of the parallel fibers (I-LTD_stim_). This form of plasticity requires the activation of NMDA receptors that contain GluN2D subunits. We therefore determined whether the GluN2D subunits of NMDA receptors are also required for extinction of fear memory.

**FIGURE 4:**
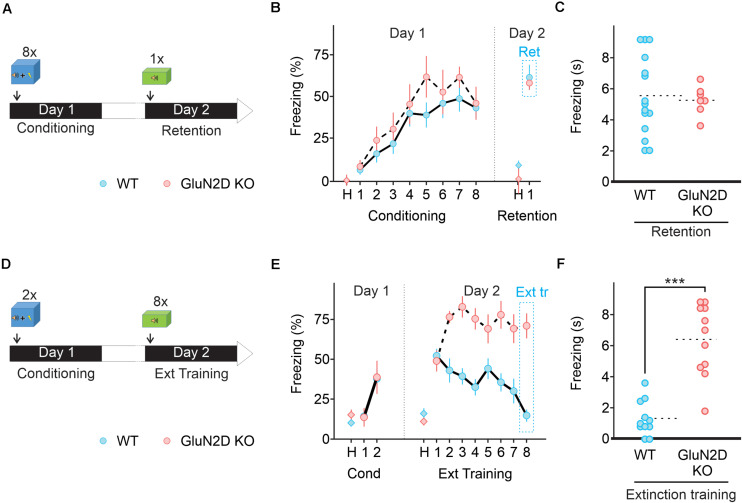
Deletion of GluN2D does not alter fear conditioning learning or memory retention but abolishes extinction learning. **(A)** Protocol used for fear conditioning. Mice were habituated for 2 min in the conditioning chamber (context A) and exposed to eight pairings of a 10 s tone that co-terminated with a 1 s foot-shock. Mice were then left in the conditioning chamber for 2 min and returned to their home cage. Next day mice were exposed to a single 10 s tone in context B. **(B)** Percentage of freezing in wildtype (blue symbols, *n* = 10) and GluN2D KO mice (red symbols, *n* = 8) during the habituation period (H), the conditioning training (tones 1–8) and retention test tone. **(C)** Freezing time in individual animals during fear memory retention test. Mean values are represented as doted lines. **(D)** A two pairing conditioning paradigm was used. Next day retention and extinction learning (Ext tr) were tested in context B by exposing the animals to eight 10 s tones after 2 min of habituation. **(E)** GluN2D KO mice (red symbols, *n* = 11), but not wildtype (blue symbols, *n* = 11), showed impaired extinction learning assessed on the last tone. **(F)** Freezing time in individual animals at the end of extinction training. Mean values are represented as doted lines. ***P < 0.001. Values in the time courses are mean ± SEM. Statistical analysis values can be found in the Supplementary Table 1.

We assessed whether the genetic deletion of GluN2D affected associative fear learning using a paradigm that consists of eight pairings of a tone (CS) co-terminated with an electric foot shock (US, Figure 4A) in context A. Both wildtype (*n* = 15) and GluN2D KO mice (*n* = 7) exhibited low freezing during the acclimation period (Figure 4B), and a similar level of total travel distance and time spent in the center, but reduced number of entries into the center square in the open field test (Supplementary Figure 2). During learning, tone-evoked freezing increased in GluN2D KO mice, which was comparable to the level observed in wildtype mice (Figure 4B). Thus, GluN2D deletion did not affect basal freezing nor fear learning acquisition. Next day, mice were tested for the retention of fear memory in context B. During the acclimation period both genotypes exhibited very little freezing (Figure 4B), indicating no fear generalization. When presented with a single tone, both wildtype and GluN2D KO mice exhibited tone-evoked freezing (Figures 4B,C). This result indicates that genetic deletion of GluN2D did not affect memory retention.

Since GluN2D was not required for learning and memory retention, we determined its role in the extinction of fear memory. Mice were subject to two pairs of tone and footshocks on day one, a learning paradigm that has been widely used to evaluate subsequent extinction learning (Figure 4D). Next day wildtype mice exhibited an increase in freezing in response to the first tone of the extinction training, reflecting a successful fear memory retention (Figure 4E). The freezing time markedly decreased to the following tones and returned to the basal level on the last tone, indicating successful extinction learning. In contrast to wildtype mice, GluN2D KO mice exhibited a high level of freezing throughout the extinction protocol, and therefore did not show any extinction learning (Figures 4E,F). A subset of mice was re-exposed to tones on day 3 (Supplementary Figure 3A) to quantify extinction memory. On the first tone wildtype mice exhibited a low level of freezing response, as observed at the end of extinction training (*n* = 4). As expected GluN2D KO mice (*n* = 5) showed an elevated level of freezing during extinction memory test, indistinguishable from that detected in original memory retention test, (Supplementary Figure 3) suggesting the inability of these mice to extinguish fear memories. These results suggest that GluN2D-containing NMDA receptors are required for the extinction of fear memories.

### D-Cycloserine and Retrieval-Extinction Paradigm Fail to Rescue Extinction Learning in GluN2D KO Mice

**FIGURE 5:**
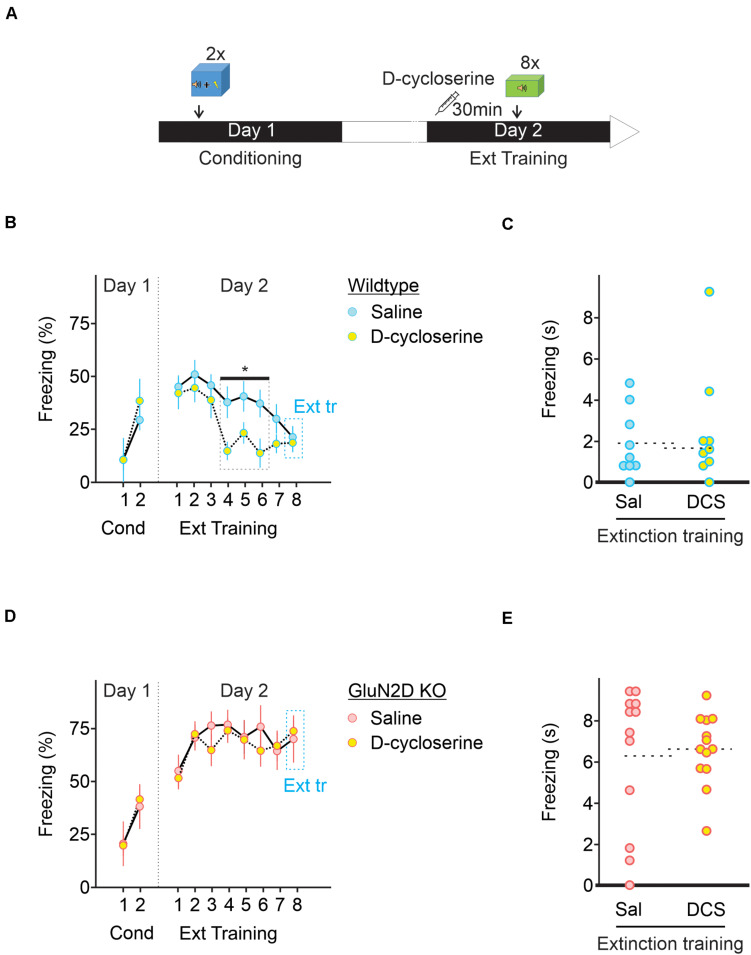
D-cycloserine fails to rescue extinction learning in GluN2D KO mice. **(A)** Wildtype and GluN2D KO mice were injected with D-cycloserine (DCS, 10 mg/kg; i.p.) or saline (Sal), 30 min before fear extinction learning. **(B)** Freezing response in wildtype mice injected with either saline (blue symbols, *n* = 9) or D-cycloserine (yellow symbols, *n* = 8) showed that D-cycloserine administration significantly accelerated extinction learning on tones 4–6. **(C)** Individual values for freezing response during extinction training. **(D)** Freezing response in GluN2D KO mice injected with either saline (red symbols, *n* = 12) or D-cycloserine (yellow symbols, *n* = 13) showed that D-cycloserine administration failed to accelerate extinction learning. **(E)** Individual freezing values at the end of the extinction training. *P < 0.05. Values are mean ± SEM. Statistical analysis values can be found in the Supplementary Table 1.

Extinction learning and retrieval behaviors are influenced by a number of pharmacological agents and behavior strategies (Pittig et al., 2016; Singewald and Holmes, 2019). One of the pharmacological agents is D-cycloserine (DCS) that binds to and potentiates NMDARs and can accelerate extinction learning and facilitates memory formation (Lee et al., 2006; Kuriyama et al., 2011; Peyrovian et al., 2019). Because deletion of GluN2D impaired extinction learning, we tested whether administration of D-cycloserine was able to reverse the deficit of extinction learning in GluN2D KO mice. Animals received an injection of saline or 10 mg/kg D-cycloserine 30 min before extinction learning (Figure 5A). We show that administration of D-cycloserine did not affect fear memory retention in wildtype animals, but accelerated extinction learning, with a marked reduction in freezing response during tones 4–6 relative to saline injected animals (*n* = 10, Figures 5B,C). However, GluN2D KO mice exhibited a high level of freezing throughout extinction training and thus D-cycloserine failed to rescue extinction learning in these mice (Figures 5D,E). Therefore genetic deletion of GluN2D attenuates the ability of D-cycloserine to facilitate extinction learning. Considering that NMDA receptors containing this subunit have higher affinity for D-cycloserine (Dravid et al., 2010; Jessen et al., 2017), these receptors may represent the site of action of D-cycloserine.

**FIGURE 6:**
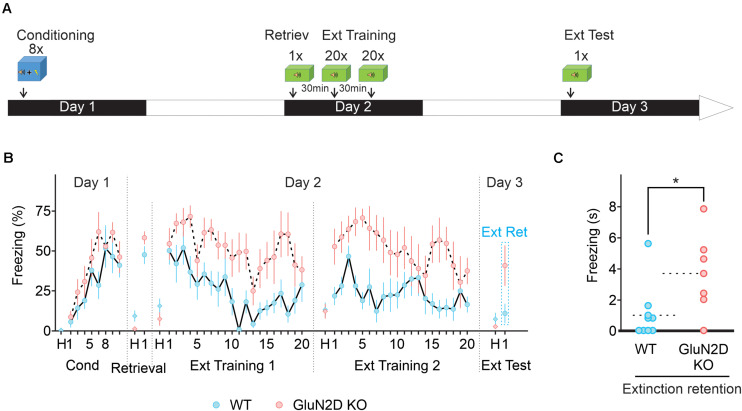
Retrieval-extinction paradigm fails to rescue extinction learning in GluN2D KO mice. **(A)** Protocol used for retrieval-extinction paradigm. Following fear conditioning, mice were exposed to a retrieval tone in context B on day 2. Thirty min later mice underwent two extinction sessions of 20 tones 30 min apart. On day 3, mice were presented with four tones in context B to test for the retention of extinction memory. **(B)** Percentage of freezing in wildtype (blue symbols, *n* = 10) and GluN2D KO mice (red symbols, *n* = 7). Wild type mice exhibited a clear extinction learning. Extinction learning in GluN2D KO mice was attenuated compared with wild type animals. **(C)** Individual freezing values at the end of extinction training. Values in the time course are mean ± SEM. *P < 0.05. Statistical analysis values can be found in the Supplementary Table 1.

One behavior strategy to enhance fear extinction is to include a retrieval trial, which is thought to initiate memory reconsolidation phase, a process that involves the cerebellum (Sacchetti et al., 2007). During the reconsolidation window extinction training can produce a persistent reduction in fear responses in mice (Monfils et al., 2009; Clem and Huganir, 2010) by destabilizing the original memory or facilitating the formation of extinction memory (Cahill and Milton, 2019). Retrieval-extinction protocol produces different effects on the expression levels of several molecular markers and patterns of Arc, compared with a standard extinction procedure (Tedesco et al., 2014; Lee et al., 2015), and may engage different neural mechanisms. We therefore determined whether extinction learning occurring during reconsolidation window when the memory becomes malleable also requires GluN2D. The extinction training consisted of one retrieval session (a single tone exposure) followed by two sessions of 20 tones (every 30 s) with a 30 min interval between the sessions (Figure 6A). In this paradigm, wildtype mice showed a marked reduction in freezing during extinction training (*n* = 10; Figure 6B). In contrast GluN2D KO mice displayed little extinction learning and there was no difference between the freezing on the first and the last tone during extinction learning (*n* = 7; Figure 6B). When extinction retention was tested on day 3 in context B, exposure to the first tone did not induce freezing in wildtype animals, indicative of a successful extinction of fear memory. However, GluN2D KO mice exhibited a high level of freezing, in response to the tone (Figures 6B,C). These results suggest that the GluN2D subunit of NMDARs is also critical for post-retrieval extinction of fear memories. Together our results that deletion of GluN2D subunits results in a strong inhibition of extinction learning reveal a new role for GluN2D-containing NMDARs in fear memory extinction.

## Discussion

**FIGURE 7:**
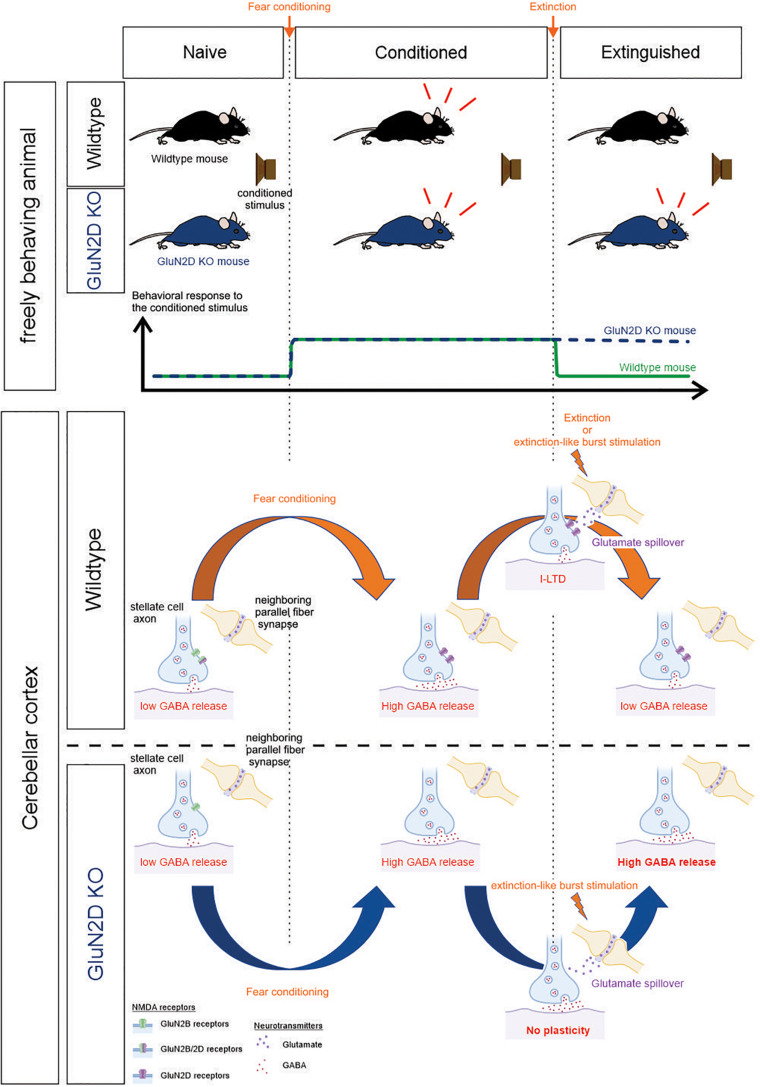
Summary schematic. ***Top***, the behavioral response of wildtype and GluN2D KO mice during fear conditioning and extinction training. ***Bottom***, correlated cellular events at the stellate-to-stellate cell synapse in the cerebellar cortex. The bottom part of the schematic was created with biorender.com.

NMDA receptors play a critical role in the extinction of associative fear memory by modulating synaptic plasticity (Dalton et al., 2012). Of the four NMDAR subunits, it has been shown that administration of a GluN2B inhibitor attenuates extinction (Sotres-Bayon et al., 2007) and GluN2C knockout impairs learning (Hillman et al., 2011). GluN2D subunits are expressed at a high level in GABAergic interneurons and are present at axon terminals where they modulate inhibitory transmission (Akazawa et al., 1994; Monyer et al., 1994; Thompson et al., 2000). GluN2D-containing NMDARs exhibit a very high affinity for glutamate and a brief activation of GluN2D receptors evokes a current with an exceedingly slow decay time. Such receptors are ideally suited for detecting the low levels of spillover glutamate that can modulate GABA release from inhibitory interneurons. We therefore determined the role of GluN2D in associative memory extinction and associated neural plasticity. Our results show that GluN2D-NMDARs are required for both extinction learning and an extinction-like stimulus-induced I-LTD_stim_ in conditioned animals and are a key component of memory extinction and associated synaptic plasticity (Figure 7). Therefore selective activation of GluN2D is a novel strategy that could enhance the extinction of fear memory.

The cerebellum is not only critically involved in the consolidation and reconsolidation of associative fear memories (Sacchetti et al., 2002, 2007; Scelfo et al., 2008; Dubois et al., 2020), but also regulates extinction learning. A recent study shows that optogenetic activation of cerebellar neurons in the fastigial nucleus that projects to vlPAG accelerates extinction learning, whereas chemogenetic inhibition of this pathway attenuates extinction (Frontera et al., 2020). Its involvement in memory extinction is further evidenced by human imaging studies (Kattoor et al., 2014; Utz et al., 2015), and impaired extinction learning of associative fear memory in mice with deletion of the L7 protein in Purkinje cells (Walton et al., 2012). Given that synaptic plasticity is the cellular substrate of learning and memory, one key question is whether extinction learning alters synaptic transmission and thereby the activity of cerebellar circuits. Here we show that while fear conditioning enhanced GABA release, extinction learning suppressed GABA release from cerebellar MLIs (I-LTD_*ext*_). This long-term depression at inhibitory synapses in the cerebellar cortex may serve as one of the cellular mechanisms underlying extinction learning. Because an auditory tone activates the mossy fiber pathway (Aitkin and Boyd, 1978) that stimulates granule cells, burst stimulation of parallel fibers (the axons of granule cells) *in vitro* would mimic the repeated exposure to the extinction stimulus *in vivo*. We have previously shown that stimulation of parallel fibers induces a lasting increase in GABA release at MLI synapses in naïve mice (Lachamp et al., 2009; Dubois et al., 2016). In contrast to naïve mice, we show that after fear conditioning parallel fiber stimulation induces a sustained decrease in GABA release (I-LTD_stim_). Thus learning enables subsequent extinction-like stimulus to induce a novel form of plasticity, and triggers synaptic metaplasticity. This form of I-LTD_stim_ mimics the one observed after fear extinction learning.

Fear conditioning increases GABA release from MLIs onto Purkinje cells and other synaptically connected MLIs (Sacchetti et al., 2004; Scelfo et al., 2008; Dubois et al., 2020). We have recently shown that fear conditioning reduces tonic endocannabinoid levels in cerebellar lobules V/VI. This is driven by increased MLI activity, as optogenetic stimulation of MLIs in naïve animals is sufficient to induce the change. A decrease in endocannabinoid signaling elevates GABA release due to dis-inhibition and is responsible for a learning-induced increase in GABA release (Dubois et al., 2020). After fear conditioning, stimulation of parallel fibers, that mimics extinction learning, induces a decrease in GABA release. Importantly deletion of GluN2D did not prevent the learning-induced increase in GABA release but abolished I-LTD_stim_ in conditioned mice. The GluN2D-dependent I-LTD_stim_ may underlie the decrease in GABA release following extinction training (Figure 7).

MLIs are spontaneously active and innervate both Purkinje cells and neighboring MLIs. Purkinje cells form inhibitory synapses onto neurons in the cerebellar nuclei, which project to other brain regions. A learning-induced increase in spontaneous GABA release could reduce Purkinje cell activity and alter their firing pattern, and consequently increase the activity of neurons in the cerebellar nuclei. Fear conditioning induces LTP at both excitatory and inhibitory synapses onto Purkinje cell (Sacchetti et al., 2004). While the former facilitates temporal summation, the enhanced feedforward inhibition serves to maintain the temporal fidelity of the cerebellar circuit (Scelfo et al., 2008). Strengthening synaptic connections among MLIs may promote the synchronized activity of inhibitory network (Bartos et al., 2007). A decrease in GABA release following extinction would be expected to reduce the output of the cerebellar circuit and fear memory.

Our result show that genetic deletion of GluN2D subunits impaired extinction learning and abolished synaptic plasticity in cerebellar MLIs. Given that the cerebellar activity can regulate extinction learning (Walton et al., 2012; Frontera et al., 2020), the deficit in cerebellar neural plasticity is likely to translate to the impaired fear extinction in GluN2D KO mice. Consistent with this model we observed a strong correlation between behavioral phenotype and associated synaptic plasticity at the cerebellar inhibitory synapse, with no effects of GluN2D KO on fear learning and I-LTP, but complete disruption of both extinction learning and I-LTD_stim_. However, other cells in the cerebellum also express GluN2D-containg NMDA receptors, including cells in the deep cerebellar nuclei and Golgi cells (Momiyama et al., 1996; Cull-Candy et al., 1997), the latter forming inhibitory synapses onto granule cells. Using a global GluN2D knockout mice, we cannot rule out possible roles of other GluN2D expressing neurons in extinction learning. Further work using a cerebellar MLI specific GluN2D KO would be required to test whether the lack of cerebellar I-LTD_stim_ causes the extinction learning impairment in mutant mice. Current models of fear extinction circuitry encompass the medial prefrontal cortex, the amygdala, and the hippocampus (Maren and Quirk, 2004; Sotres-Bayon et al., 2004, 2007; Ogden et al., 2014). Since GluN2D subunits are also found in interneurons in the cortex, thalamus and hippocampus (Monyer et al., 1994; Standaert et al., 1996; Yamasaki et al., 2014; von Engelhardt et al., 2015; Alsaad et al., 2019), a GluN2D-dependent plasticity in inhibitory transmission in these brain regions may also contribute to extinction learning. Dopaminergic neurons in the substantia nigra are known to express GluN2D/2B triheteromeric receptors at synaptic and extra-synaptic sites and deletion of GluN2D subunits reduced agonist sensitivity (Jones and Gibb, 2005; Brothwell et al., 2008; Huang and Gibb, 2014; Wild et al., 2015; Morris et al., 2018). Activation of these neurons during fear extinction had no effect on acquisition of extinction, but enhanced fear extinction memory and blocked the renewal of fear in a novel context (Bouchet et al., 2018). It would be interesting to determine whether deletion of GluN2D receptors in these dopaminergic neurons influence fear extinction memory, although they are unlikely to contribute to extinction learning. Thus, the impaired fear extinction we observed may arise because of its effects on neural plasticity in multiple brain regions, including the cerebellum and similar metaplasticity may also occur in other brain regions.

Effects of genetic deletion of GluN2D subunits on emotional behaviors have been investigated in a number of studies (Ikeda et al., 1995; Miyamoto et al., 2002; Yamamoto et al., 2017; Shelkar et al., 2019; Salimando et al., 2020). They produce conflicting results, as global GluN2D KO mice show no difference in anxiety tests (Ikeda et al., 1995), but increased immobility in the forced swim test in another study (Shelkar et al., 2019). We did not detect any difference in basal freezing in the conditioning chamber (Figures 4, 5, 6) and time spent in the center square of the open field test (Supplementary Figure 1), and this is consistent with no elevated generalized fear in mutant mice. These mutant mice exhibit lower susceptibility to stress induced by the elevated plus-maze, light–dark box, and forced swimming tests, and reduced locomotor activity in a novel environment, which is associated with altered monoamine neurotransmitter levels in several brain regions (Miyamoto et al., 2002). GluN2D receptors are also required for the antidepressant effects of ketamine (Sapkota et al., 2016; Ide et al., 2017), and mediate a stress-induced changes in cognitive function, as GluN2D KO mice show social stress-induced anhedonia and a deficit in social recognition/memory and spatial memory acquisition (Yamamoto et al., 2017). In this study we demonstrate that GluN2D-receptors mediate extinction learning of associative fear memory. This is unlikely to be attributable to a change in locomotor function as basal freezing before conditioning and total travel distance in the open field test were unaltered in mutant mice, consistent with the observation of normal rotarod motor performance (Yamamoto et al., 2013). A 10–30% decrease in total travel distance reported in GluN2D KO mice (Ikeda et al., 1995; Shelkar et al., 2019) cannot account for a fourfold higher freezing time in response to tones at the end of extinction training relative to wildtype mice (Figures 4–6), although what causes the conflicting results is unclear. A recent study show that GluN2D regulates emotional behavior in a region-specific manner as deletion of GluN2D from CRF neurons in the BNST mice increases depressive-like behaviors (Salimando et al., 2020). Our results provide further evidence for an important role of GluN2D NMDARs in emotional learning and behavior.

NMDAR subtypes that are expressed in diverse neuronal populations are involved in different aspects of learning and memory. D-cycloserine has been widely used for the treatment of fear-related pathologies (Otto et al., 2016), and was thought to act on GluN2C receptors (Ogden et al., 2014) due to its higher efficacy at GluN2C-containing NMDARs compared to GluN2A and 2B NMDARs (Sheinin et al., 2001; Dravid et al., 2010). However, we identified GluN2D-containing NMDARs as being responsible for extinction learning. Interneurons that express GluN2D subunits are likely to express GluN2B/D tri-heteromeric receptors in wildtype mice and GluN2B di-heteromeric receptors in the KO mice, as previously shown in cerebellar interneurons and dopaminergic neurons in the substantia nigra (Brickley et al., 2003; Huang and Gibb, 2014; Dubois et al., 2016). As a consequence this may reduce the ability of D-cycloserine to bind to NMDA receptors in these neurons, and thus decrease its efficacy in accelerating extinction learning. Alternatively, deletion of the GluN2D subunit may render extinction learning an NMDAR-independent process. Our finding that GluN2D receptors are critical for extinction learning highlights the need to develop pharmacological tools that selectively target this NMDAR subtype in the treatment of anxiety disorders.

## Data Availability Statement

The original contributions presented in the study are included in the article/Supplementary Material, further inquiries can be directed to the corresponding author.

## Ethics Statement

The animal study was reviewed and approved by IACUC.

## Author Contributions

CJD performed experiments and analysis. CJD and SJL designed the study and wrote the manuscript. Both authors contributed to the article and approved the submitted version.

## Conflict of Interest

The authors declare that the research was conducted in the absence of any commercial or financial relationships that could be construed as a potential conflict of interest.

## Footnotes

1 http://mousegenotype.com

2 https://www.estimationstats.com
