# Supplementary material for: GluN2D NMDA Receptors Gate Fear Extinction Learning and Interneuron Plasticity
Source: Front Synaptic Neurosci. 2021 May 24;13:681068. doi: 10.3389/fnsyn.2021.681068 (PMC8183684; doi:10.3389/fnsyn.2021.681068)
Supplement: Supplementary file 1 [file Data_Sheet_1.PDF]

Fig	Parameter analyzed	Conditions	n	Analysis	*F* value	*P* value
**1C**	mIPSC frequency in WT	N *vs* FC	8 - 9	Mann-Whitney	*F* (1,30)= 4.936	**0,006**
		N *vs* Ext	8 - 6	Mann-Whitney		0,95
		Ext *vs* FC	6 - 9	Mann-Whitney		**0,039**
	mIPSC amplitude in WT	N *vs* FC	8 - 9	Mann-Whitney		0,532
		N *vs* Ext	8 - 6	Mann-Whitney		0,662
		Ext *vs* FC	6 - 9	Mann-Whitney		0,517
**3B**	mIPSC frequency in WT	N *vs* FC	11 - 10	Mann-Whitney		**0,007**
	mIPSC amplitude in WT	N *vs* FC	11 - 10	Mann-Whitney		0,86
**Text (Fig2/3)**	mIPSC frequency	conditioning *vs* genotype		2 WAY ANOVA	*F* (1,37)= 0.384	0,54
		genotype		1 WAY ANOVA	*F* (1,37)= 0.343	0,562
		conditioning		1 WAY ANOVA	*F* (1,37)= 14.083	**<0,001**
**3G**	mIPSC frequency in WT	Pre *vs* post	5	Wilcoxon		**0,007**
	mIPSC frequency in GluN2D	Pre *vs* post	6	Wilcoxon		>0.999
	mIPSC ampitude in WT	Pre *vs* post	5	Wilcoxon		0,438
	mIPSC amplitude in GluN2D	Pre *vs* post	6	Wilcoxon		0,563
**4C**	Freezing time - habituation day 1	WT vs GluN2D KO	15 - 7	Mann-Whitney		0,646
	Freezing time - conditioning	WT vs GluN2D KO	15 - 7	Mann-Whitney		0,944
	Freezing time - habituation day 2	WT vs GluN2D KO	15 - 7	Mann-Whitney		0,214
	Freezing time - retention	WT vs GluN2D KO	15 - 7	Mann-Whitney		0,832
**4F**	Freezing time - conditioning	WT vs GluN2D KO	11 - 11	Mann-Whitney		0,767
	Freezing time - retention	WT vs GluN2D KO	11 - 11	Mann-Whitney		0,921
	Freezing time - extinction learning	WT vs GluN2D KO	11 - 11	Mann-Whitney		**<0,001**
	Freezing time during extinction learning	extinction *vs* genotype	11 - 11	2 WAY ANOVA RM	*F* (7,175)= 5,682	**<0,05**
**5B/C**	Freezing time during extinction learning	extinction *vs* drug injection	9 - 8	2 WAY ANOVA RM	*F* (7,167)= 0,837	>0,05
	Freezing time during extinction learning	drug effect	9 - 8	1 WAY ANOVA	*F* (1,167)=5,719	**<0,05**
	Freezing tone 1	WT Sal *vs* DCS	9 - 8	Tukey		0,73217
	Freezing tone 2	WT Sal *vs* DCS	9 - 8	Tukey		0,56117
	Freezing tone 3	WT Sal *vs* DCS	9 - 8	Tukey		0,52643
	Freezing tone 4	WT Sal *vs* DCS	9 - 8	Tukey		**0,0277**
	Freezing tone 5	WT Sal *vs* DCS	9 - 8	Tukey		**0,03512**
	Freezing tone 6	WT Sal *vs* DCS	9 - 8	Tukey		**0,00546**
	Freezing tone 7	WT Sal *vs* DCS	9 - 8	Tukey		0,22841
	Freezing tone 8	WT Sal *vs* DCS	9 - 8	Tukey		0,74536
	Freezing time - conditioning	WT Sal vs DCS	9 - 8	Mann-Whitney		0,885
	Freezing time - retention	WT Sal vs DCS	9 - 8	Mann-Whitney		0,736
	Freezing time - extinction learning	WT Sal vs DCS	9 - 8	Mann-Whitney		>0.999
**5D/E**	Freezing time - conditioning	GluN2D Sal vs DCS	9 - 8	Mann-Whitney		0,68
	Freezing time - retention	GluN2D Sal vs DCS	9 - 8	Mann-Whitney		0,624
	Freezing time - extinction learning	GluN2D Sal vs DCS	9 - 8	Mann-Whitney		0,496
**6B/C**	Freezing time - conditioning	WT vs GluN2D KO	10 - 7	Mann-Whitney		0,845
	Freezing time -Retrieval	WT vs GluN2D KO	10 - 7	Mann-Whitney		0,156
	Freezing time - retention	WT vs GluN2D KO	10 - 7	Mann-Whitney		0,807
	Freezing time - extinction learning	WT vs GluN2D KO	10 - 7	Mann-Whitney		**0,0271**
	Freezing time - extinction retention	WT vs GluN2D KO	10 - 7	Mann-Whitney		**0,0281**
**S1**	Freezing time - conditioning	WT Sal vs Mem	9 - 10	Mann-Whitney		0,178
	Freezing time - retention	WT Sal vs Mem	9 - 10	Mann-Whitney		0,595
	Freezing time - extinction learning	WT Sal vs Mem	9 - 10	Mann-Whitney		**<0,001**
**S2**	Distance traveled	WT vs GluN2D KO	7 - 13	Mann-Whitney		0,874
	Time in center	WT vs GluN2D KO	7 - 13	Mann-Whitney		0,579
	Entries in center	WT vs GluN2D KO	7 - 13	Mann-Whitney		**0,0293**
**S3**	Freezing time - retention	WT vs GluN2D KO	4 - 5	Mann-Whitney		0,903
	Freezing time - retention	WT vs GluN2D KO	4 - 5	Mann-Whitney		0,176
	Freezing time - Extinction training	WT vs GluN2D KO	4 - 5	Mann-Whitney		**0,0365**
	Freezing time - Extinction retention	WT vs GluN2D KO	4 - 5	Mann-Whitney		**0,0463**

Supplementary Table 1. Statistics table

Bold P values indicate a significant effect (P<0.05). WT, wildtype mice; GluN2D KO, GluN2D KO mice; N, Naive group; FC, paired group; Ext, extinction group; RM ANOVA, repeated measurement ANOVA.
